# AKR7A3 suppresses tumorigenicity and chemoresistance in hepatocellular carcinoma through attenuation of ERK, c-Jun and NF-κB signaling pathways

**DOI:** 10.18632/oncotarget.12726

**Published:** 2016-10-18

**Authors:** Raymond Kwok Kei Chow, Sarah Tsz-Kwan Sin, Ming Liu, Yan Li, Tim Hon Man Chan, Yangyang Song, Leilei Chen, Dora Lai-Wan Kwong, Xin-Yuan Guan

**Affiliations:** ^1^ Department of Clinical Oncology, Li Ka Shing Faculty of Medicine, the University of Hong Kong, Hong Kong; ^2^ Centre for Cancer Research, Li Ka Shing Faculty of Medicine, the University of Hong Kong, Hong Kong; ^3^ State Key Laboratory for Liver Research, Li Ka Shing Faculty of Medicine, the University of Hong Kong, Hong Kong; ^4^ School of Basic Sciences, Guangzhou Medical University, Guangzhou, China; ^5^ Department of Biology, South University of Science and Technology of China, Shenzhen, China; ^6^ Cancer Science Institute of Singapore, National University of Singapore, Singapore

**Keywords:** AKR7A3, HCC, tumor suppressor, methylation, chemoresistance

## Abstract

Hepatocellular carcinoma (HCC), which accounts for 85–90% of primary liver cancer, is now the second leading cause of cancer-related mortality worldwide. Here we reported that Aldo-Keto Reductase family 7A isoform 3 (AKR7A3) is frequently down-regulated in HCC, associating with poor overall survival rate, elevated serum α-fetoprotein (AFP) and poor differentiation of HCC. The promoter region of AKR7A3 was detected to be hypermethylated. Loss of heterozygosity (LOH) was also detected in AKR7A3. Functional assays on both AKR7A3 overexpressed and knockdown cells, including foci formation, colony formation in soft agar, migration, invasion and tumor formation in nude mice, demonstrated the strong tumor suppressive functions of AKR7A3. In addition, treatment of chemotherapy drug cisplatin showed that AKR7A3 sensitizes tumor cells to apoptosis. Mechanistically, western blot analysis showed that overexpression of AKR7A3 inhibits the activation of ERK, c-Jun and NF-κB. In summary, we found that AKR7A3 functions as a tumor suppressor gene in HCC through attenuating c-Jun, ERK and NF-κB signaling pathways.

## INTRODUCTION

Hepatocellular carcinoma (HCC) is one of the most frequently diagnosed malignancies with poor prognosis [1]. It is widely accepted that abnormal chromosomal changes, including amplification and deletion, greatly contribute to the onset of HCC [2]. Accumulation of genetic alterations may lead to constant activation of proto-oncogenes and inactivation of important tumor suppressor genes, resulting in abnormalities in cell functions such as cell proliferation and apoptosis [3]. In HCC, some specific chromosomal regions were found to be frequently deleted. These regions include 1p, 4q, 8p, 13q, 16q and 17p [4–5].

Aldo-Keto Reductase Family 7, Member A3 (AKR7A3), which locates on chromosome 1p36, belongs to the aldo-keto reductases (AKRs) superfamily. This super family consists of 15 families with more than 140 members, which function to reduce aldehydes and ketones to alcohols [6]. These enzymes were also reported to play important roles in nuclear receptor signaling, cellular metabolism, inflammatory responses, osmoregulation, endobiotic and xenobiotic detoxification and hormone synthesis [6–7]. Accumulating evidence supported that AKR1 and AKR7 are involved in the development of cancers such as breast, lung, liver, colorectal and prostate cancers [8]. AKR7A3 is responsible for the detoxification of aflatoxin B1—a potent hepatocarcinogen [9]. In breast cancer, high expression level of AKR7A3 significantly associates with longer disease free survival [10].

Here, we reported that AKR7A3 is frequently down-regulated in HCC tissues compared to adjacent non-tumor tissues. Hypermethylation of promotor regions and chromosome deletion are found to be responsible for the down-regulation of AKR7A3. Clinically, low AKR7A3 expression is associated with poor overall survival, elevated serum AFP and poor differentiation of HCC tissue, indicating the important role of AKR7A3 in the suppression of HCC. *In vitro* functional studies, including foci formation, colony formation in soft agar, migration and invasion, revealed strong tumor suppressive functions of AKR7A3. *In vivo* tumor formation experiments in nude mice also demonstrated the inhibition of tumor formation by AKR7A3 overexpression. Downstream factors of EMT markers and signaling pathways crucial in cancer development including ERK, c-Jun and NF-κB were also detected. Western blot results demonstrated that AKR7A3 could inhibit the phosphorylation of ERK, c-Jun and NF-κB in HCC cell lines.

## RESULTS

### The clinical significance of AKR7A3

**Figure 1:**
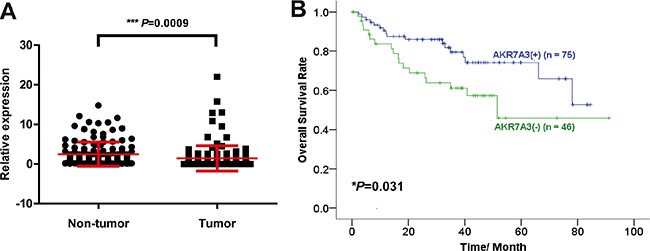
AKR7A3 is frequently down-regulated in HCC with clinical significance (**A**) Expression level of AKR7A3 in 129 pairs of HCC tumor and adjacent non-tumor samples detected by qRT-PCR. ***P = 0.0009. (**B**) Overall survival rate of AKR7A3 normal expression (+) and low expression (−) patients. *P = 0.031.

Three pairs of primary HCC tumor samples and adjacent non-tumor tissues were subjected to transcriptome sequencing. Sequencing data revealed that AKR7A3 was among the 102 genes that were down-regulated in all the 3 HCC samples, as compared to paired non-tumor samples. To detect the expression level of AKR7A3 in a larger cohort of HCC samples, qRT-PCR was performed on 129 pairs of HCC patient samples. Results showed AKR7A3 was significantly down-regulated in HCC (***P = 0.0009) (Figure 1A). In 50 out of 129 tested sample pairs, AKR7A3 was detected with more than 2 folds of down-regulation. Western blot analysis on 10 pairs of HCC samples also supported the above results, where AKR7A3 expression significantly reduced in HCC tissues (Supplementary Figure S1). The down-regulation of AKR7A3 was also significantly associated with poor overall survival rate (*P = 0.031) (Figure 1B), elevated serum AFP level (***P < 0.001) (Table 1) and poor differentiation of HCC (*P = 0.011) (Table 1).

**Table 1:** Association of AKR7A3 down-regulation with clinicopathological features in 129 primary HCCs

Features	Total	AKR7A3 expression				*P* value
		Normal expression		Down-regulation		
**Gender**						
Male	106	68	(64.2%)	38	(35.9%)	
Female	23	11	(47.8%)	12	(52.2%)	0.162
**Age**						
≤ 60	105	61	(58.1%)	44	(41.9%)	
> 60	24	18	(75%)	6	(25%)	0.165
Hepatitis B surface antigen*						
Positive	96	57	(59.4%)	39	(40.6%)	
Negative	25	19	(76%)	6	(24%)	0.165
**Serum α-fetoprotein level (ng/ml)**						
< = 400	64	50	(78.1%)	14	(21.9%)	
> 400	56	25	(44.6%)	31	(55.4%)	**< 0.001**
**Cirrhosis**						
Present	85	55	(64.7%)	30	(35.3%)	
Absent	36	21	(58.3%)	15	(41.7%)	0.541
**Tumor stage**						
Stage I	86	54	(62.8%)	32	(37.2%)	
Stage II	5	3	(60%)	2	(40%)	
Stage III	30	17	(56.7%)	13	(43.3%)	0.838
**Tumor size**						
< 5 cm	38	20	(52.6%)	18	(47.4%)	
> 5 cm	85	56	(65.9%)	29	(34.1%)	0.228
**Differentiation**						
Well differentiated (I-II)	66	49	(74.2%)	17	(25.8%)	
Moderately differentiated (II-III)	44	21	(47.7%)	23	(52.3%)	
Poorly differentiated (III-IV)	3	1	(33.3%)	2	(66.7%)	**0.011**
**Metastasis**						
Positive	59	37	(62.7%)	22	(37.3%)	
Negative	70	42	(60%)	28	(40%)	0.856

*Partial data was not available, and the statistical analysis was based on available data.

### Promotor hypermethylation and allele loss contribute to AKR7A3 down-regulation in HCC

**Figure 2:**
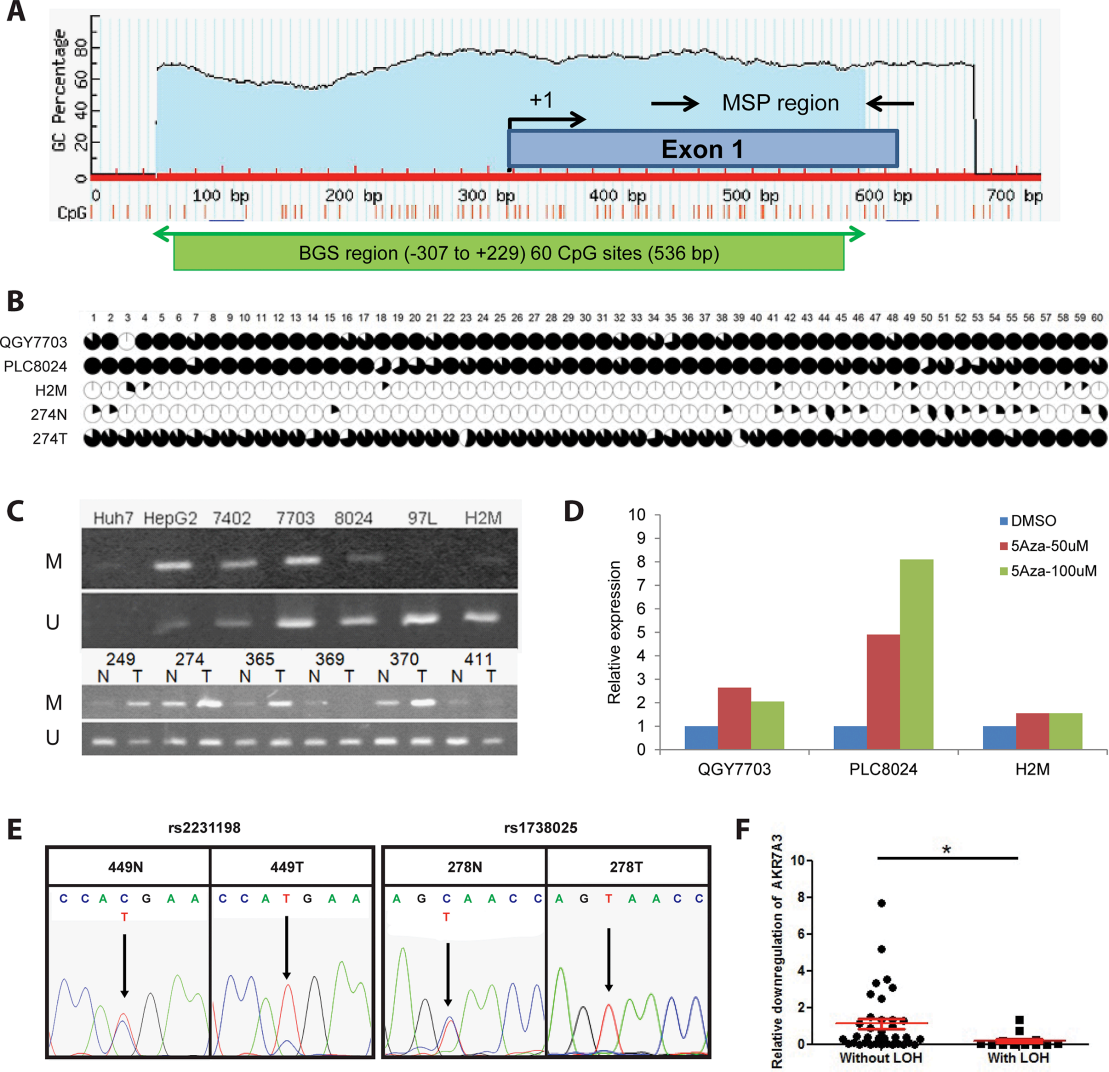
Promoter hypermethylation and LOH of AKR7A3 were observed in HCC (**A**) A CpG island (−307 to +229) of 536 bp covering 60 CpG sites were predicted by software MethPrimer. BGS and MSP regions are indicated. (**B**) Methylation status of promoter region of AKR7A3. The areas of black regions within each circle indicate the percentage of colonies with the specific CpG site methylated. HCC cell lines of low (QGY7703 and PLC8024) and high (H2M) AKR7A3 expression, as well as paired human HCC tissue samples, including non-tumor and tumor samples, were examined. (**C**) Methylation-specific PCR results of HCC cell lines and clinical samples. DNA extracted from cell lines and clinical samples were detected by MSP using methylation-specific primers (M: methylated; U: unmethylated). (**D**) The expression level of AKR7A3 in cell lines with low (QGY7703 and PLC8024) or high (H2M) AKR7A3 expression after 50 uM or 100 uM of 5-Aza-dC and vehicle control treatment. (**E**) Representative images showing DNA samples with LOH, indicated by the presence of SNP dual peaks in non-tumor samples (449N, 278N) and single peaks in tumor samples (449T, 278T) at 2 SNP (rs2231198 and rs1738025) positions. (**F**) Comparison of fold change of AKR7A3 expression between patient samples with or without LOH. *P < 0.05.

Epigenetic alternations, including promoter hypermethylation, is one of the most frequent causes of gene down-regulation [11]. In order to investigate the reasons behind AKR7A3 down-regulation, the methylation status of AKR7A3 promotor region was studied. A 536 bp CpG island within the AKR7A3 promoter region containing 60 CpG sites was predicted using MethPrimer software (Figure 2A). Primers were designed to amplify this region from the bisulfite treated DNA of HCC 3 cell lines and one pair of HCC primary samples. In cell line samples, endogenous AKR7A3 expression was first detected by qRT-PCR (Supplementary Figure S2), followed by BGS analysis, which results showed that the methylation frequency was significantly higher in HCC cell line with low AKR7A3 expression (QGY7703 and PLC8024) than that in the cell line with high AKR7A3 expression (H2M) (Figure 2B). In paired HCC samples (274N and 274T), where AKR7A3 was down-regulated by more than 100 folds in tumor tissue, the promotor region of AKR7A3 was found to be hypermethylated in tumor tissue, as compared to adjacent non-tumor tissue (Figure 2B).

MSP was subsequently performed to detect the methylation status in a larger amount of samples. Our results showed that methylated allele was more prominent in AKR7A3 low-expressing cell lines (Huh7, HepG2, BEL7402, QGY7703 and PLC8024), while unmethylated allele was more prominent in AKR7A3 high-expressing cell lines (97L and H2M) (Figure 2C, upper panel). Analysis on 79 HCC DNA detected methylated allele in 33/79 (52%) of HCC cases (Table 2). The frequency of AKR7A3 promoter hypermethylation with AKR7A3 down-regulation was significantly higher (16/21, 76%) than that in HCC cases without down-regulation (*P = 0.011) (Table 2). Representative images of six paired HCC samples with AKR7A3 down-regulation showed higher prominence of methylated allele in tumor samples and unmethylated allele in non-tumor samples (Figure 2C, lower panel).

**Table 2:** The correlation between AKR7A3 methylation and expression level of AKR7A3 in HCC patients

		AKR7A3 down-regulation		Total	*P* value
		Absent	Present		
**AKR7A3 promoter**	Absent	33 (57%)	25 (43%)	58 (73%)	**0.011**
**methylation**	Present	5 (24%)	16 (76%)	21 (27%)	
	Total	46 (48%)	33 (52%)	79	

To further evaluate whether AKR7A3 down-regulation is directly affected by the methylation of AKR7A3 promoter, three HCC cell lines with low (QGY7703 and PLC8024) and high (H2M) AKR7A3 expression were treated with gradient concentrations of demethylating agent 5-azacytidine (5-aza-dC). After treatment, AKR7A3 expression was detected by qRT-PCR. Results showed that 5-aza-dC treatment successfully restored AKR7A3 expression in both cell lines with promoter hypermethylation (QGY7703 and PLC8024) but not in the cell line without promoter hypermethylation (H2M) (Figure 2D).

In HCC, chromosome 1p is frequently detected with chromosome material loss [5]. Our transcriptome sequencing data revealed 19 down-regulated genes that are located on chromosome 1p, AKR7A3 being one of them. The fact that AKR7A3 locates in this region leads us to look into whether chromosome loss plays a role in the down-regulation of AKR7A3. To test this hypothesis, two sets of primers flanking 2 SNP positions (rs1738025 and rs2231198, located on exon 2 and exon 6 of AKR7A3, respectively) were designed to amplify two regions within AKR7A3 coding sequence. The PCR product was sequenced and dual peaks at either one of the two SNPs were found in 55 out of 85 of the tested non-tumor DNA samples (Figure 2E). Among these 55 samples, 30 showed AKR7A3 down-regulation and 13 of them were detected with allele loss, demonstrated by LOH of tumor DNA samples (Table 3). The AKR7A3 expression levels were further compared between HCC cases with and without LOH. The relative down-regulation of AKR7A3 in HCC samples with LOH was significantly more aggravated than that without LOH (*P < 0.05) (Figure 2F). These results demonstrated the important role of chromosome deletion in the down-regulation of AKR7A3.

**Table 3:** The correlation between LOH status and expression level of AKR7A3 in HCC patients

		LOH at AKR7A3 region		Total	*P* value
		Absent	Present		
**AKR7A3 expression**	Normal	20 (80%)	5 (20%)	25 (45%)	0.087
	Down-regulated	17 (57%)	13 (43%)	30 (55%)	
	Total	37 (67%)	18 (33%)	55	

### AKR7A3 demonstrated strong tumor suppressive functions

**Figure 3:**
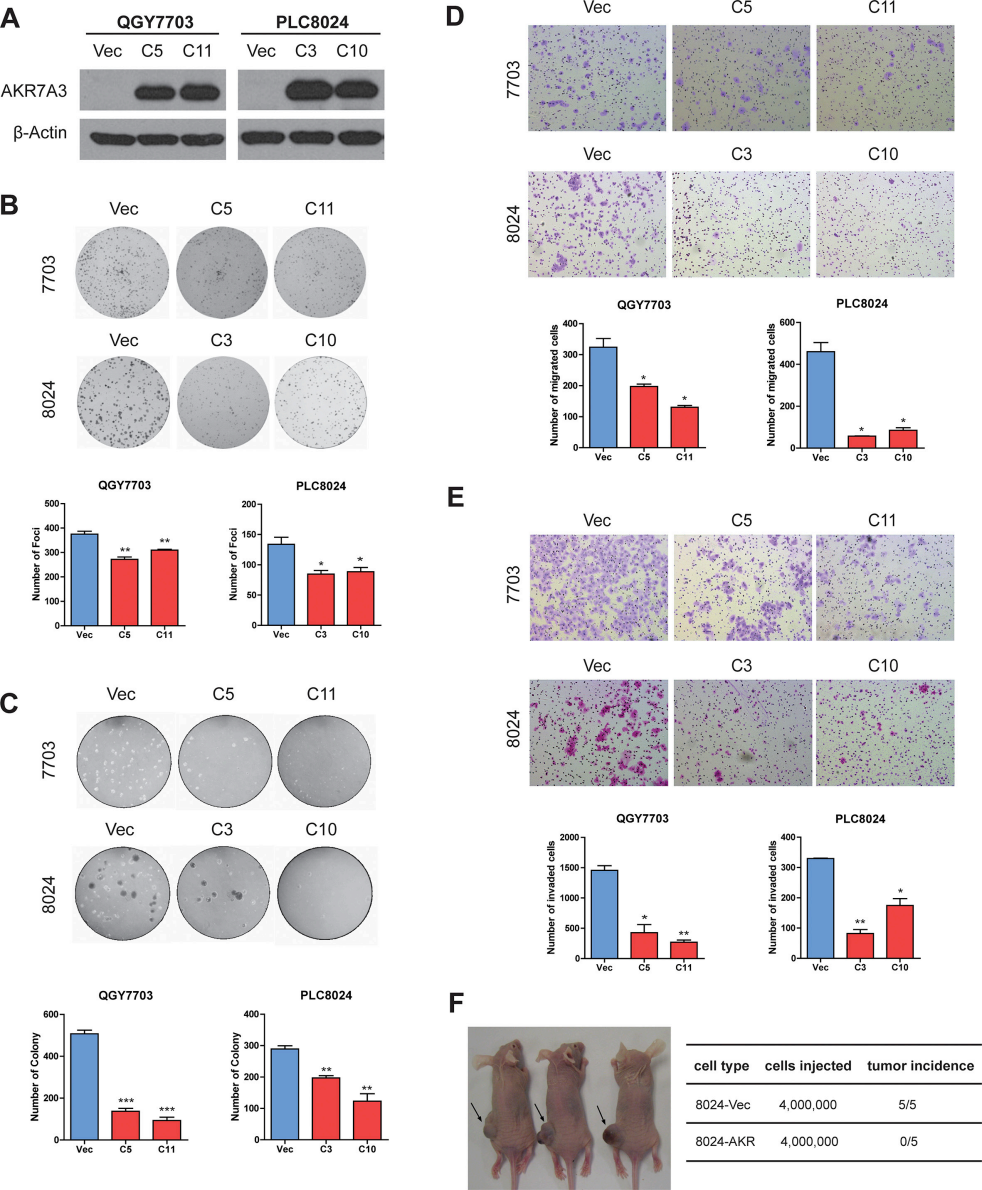
AKR7A3 demonstrated strong tumor suppressive functions (**A**) Western blot showing successful overexpression of AKR7A3 in QGY7703 and PLC8024 cell lines. β-actin was used as loading control. (**B**) Representative images and quantification of number of foci formed by QGY7703 and PLC8024 vector and AKR7A3 transfected cells. *P < 0.05, **P < 0.01. (**C**) Representative images and quantification of number of colonies formed in soft agar assay by QGY7703 and PLC8024 vector and AKR7A3 transfected cells. **P < 0.01, ***P < 0.001. (**D**) Representative images and quantification of migrated cells and (**E**) invaded cells of QGY7703 and PLC8024 vector and AKR7A3 transfected cells. *P < 0.05, **P < 0.01. (**F**) Representative image and quantification of *in vivo* tumor formation assay of PLC8024 vector and AKR7A3 transfected cells on nude mice.

In order to study the tumor suppressive functions of AKR7A3 in HCC, the CDS sequence of this gene was cloned into a lentiviral vector. Cells transfected with empty vector were used as controls. Blasticidin was applied to the transfected cells to screen for two stably transfected cells in two cell lines (QGY7703 and PLC8024) with relatively low endogenous AKR7A3 expression (Supplementary Figure S2). For each cell line, two clones with highly expressed AKR7A3 were selected for further studies (Figure 3A). The tumor suppressive functions of AKR7A3 *in vitro* were investigated by foci formation, soft agar, migration and invasion assays. In cells transfected with AKR7A3, significantly lower number of foci formed (*P < 0.05; **P < 0.01) (Figure 3B) and colonies formed in soft agar (**P < 0.01; ***P < 0.001) (Figure 3C) were detected, as compared with control cells. In migration and invasion assays, AKR7A3 transfected cells demonstrated significantly lower ability to migrate (*P < 0.05) (Figure 3D) and invade (*P < 0.05; **P < 0.01) (Figure 3E). We further validated AKR7A3 functions through *in vivo* tumor formation assay in nude mice. PLC8024-AKR7A3 and control cells were subcutaneously injected into the right and left dorsal flanks of 5 nude mice, respectively. PLC8024-AKR7A3 could not form tumor in any of the 5 mice, whereas PLC8024-vector cells formed tumor in all mice in the experiment (Figure 3F).

### AKR7A3 knockdown enhances oncogenicity of HCC

**Figure 4:**
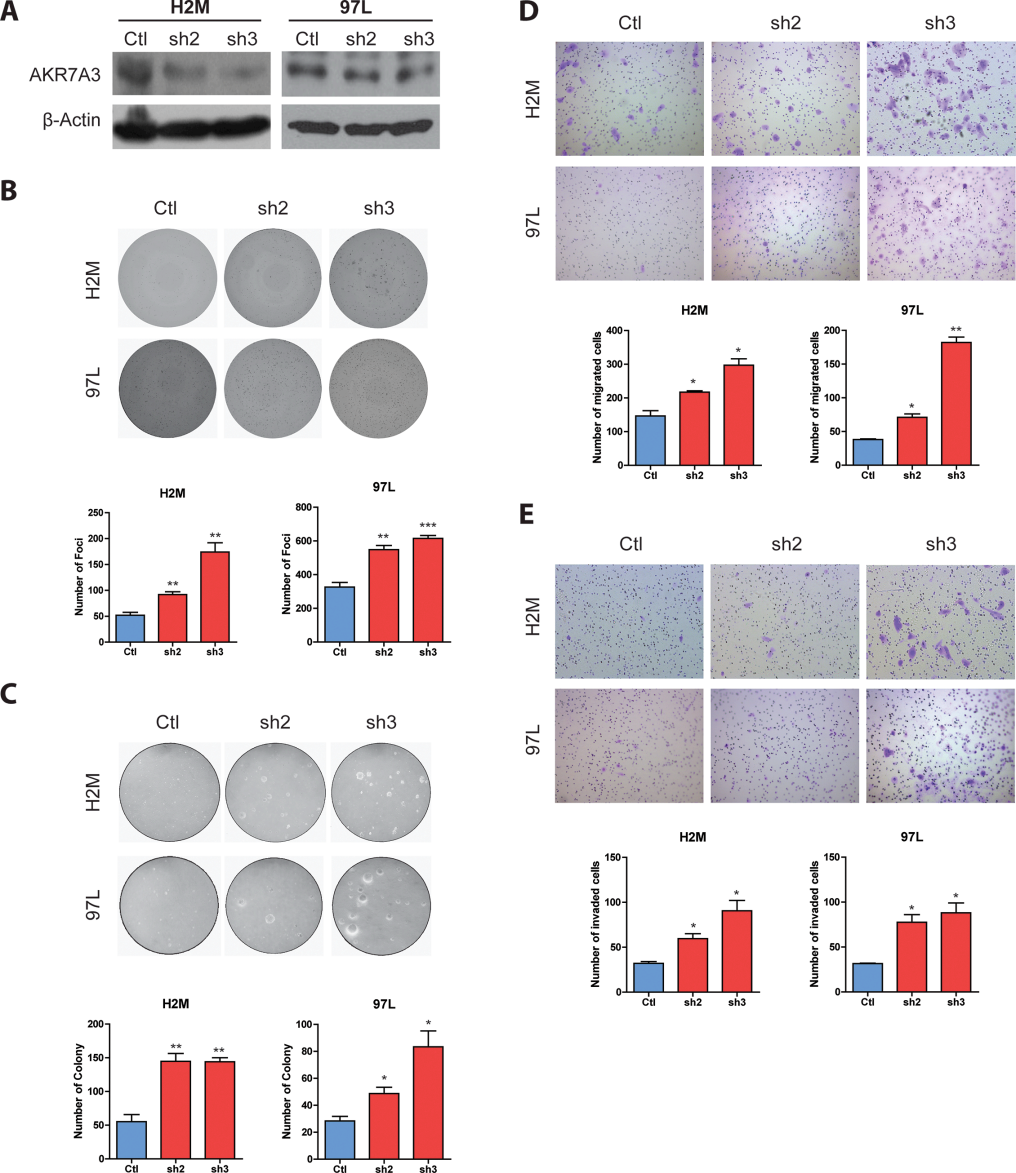
Knockdown of AKR7A3 induced tumorogenicity characteristics of HCC cell lines (**A**) Western blot showing successful knockdown of AKR7A3 in H2M and 97L cell lines. β-actin was used as loading control. (**B**) Representative images and quantification of foci formed by H2M and 97L vector and shRNA transfected cells. **P < 0.01, ***P < 0.001. (**C**) Representative images and quantification of colonies formed in soft agar assay by H2M and 97L vector and shRNA transfected cells. *P < 0.05, **P < 0.01. (**D**) Representative images and quantification of migrated and (**E**) invaded cells of H2M and 97L vector and shRNA transfected cells. *P < 0.05, **P < 0.01.

To further access the effect of AKR7A3 down-regulation, two HCC cell lines (H2M, 97L) with relatively high endogenous AKR7A3 expression was stably transfected with two short hairpin RNAs (shRNA) targeting AKR7A3. The knockdown of AKR7A3 was confirmed by western blot (Figure 4A). Vector cloned with scrambled shRNA was used as control. In foci formation and soft agar assays, AKR7A3 knockdown cells formed significantly higher numbers of colonies, as compared to control cells (*P < 0.05; **P < 0.01; ***P < 0.001) (Figure 4B and 4C). AKR7A3 knockdown cells also showed stronger abilities to migrate (*P < 0.05; **P < 0.01) (Figure 4D) and invade (*P < 0.05) (Figure 4E).

### AKR7A3 inhibits chemoresistance of HCC

**Figure 5:**
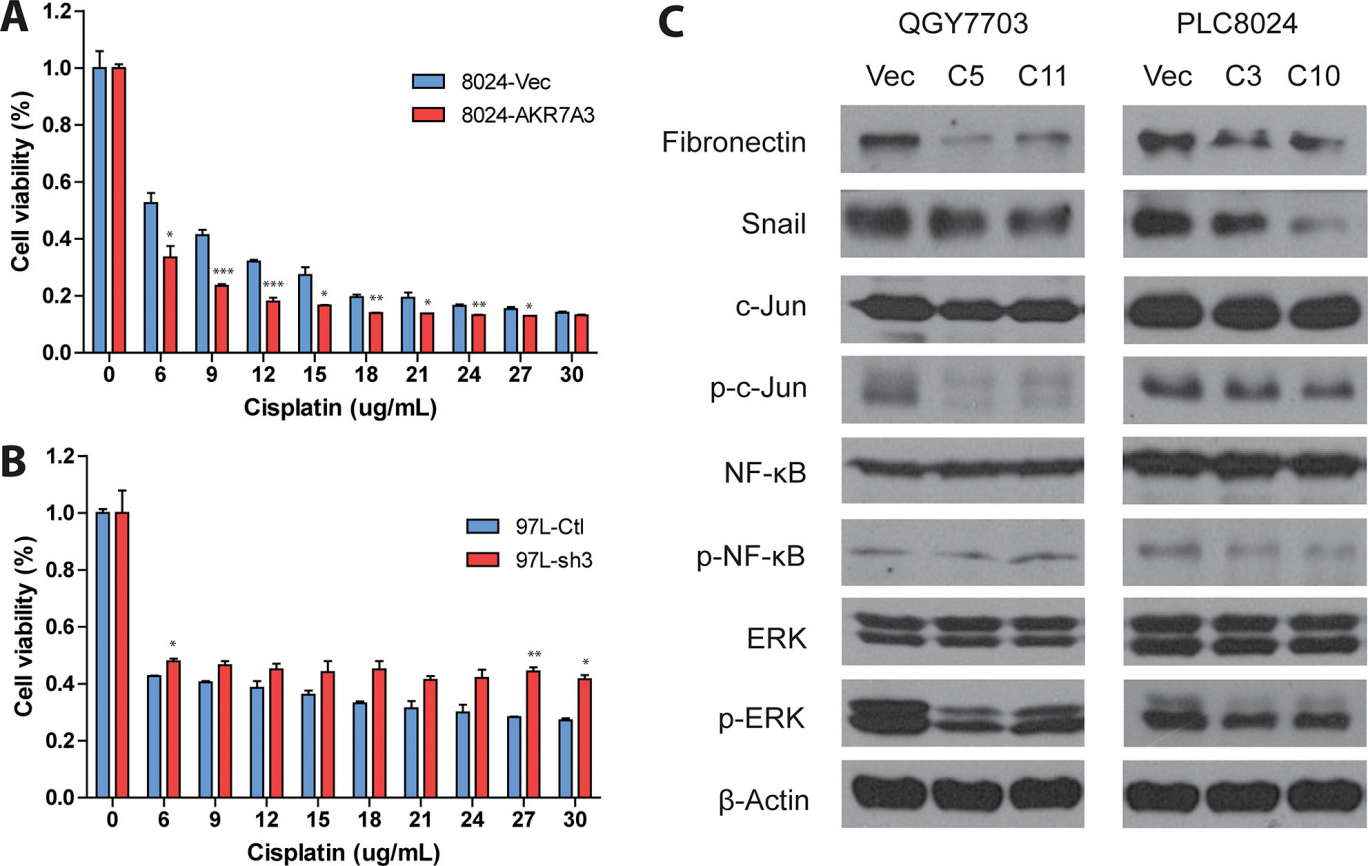
AKR7A3 overexpressed cells are more sensitive to cisplatin and showed lower activity of ERK, c-Jun and NF-κB (**A**) Cell viability assay of PLC8024 vector or AKR7A3 overexpressed cells after treatment by gradient concentrations of cisplatin. *P < 0.05, **P < 0.01, ***P < 0.001. (**B**) Cell viability assay of 97L control or shRNA transfected cells after treatment by gradient concentrations of cisplatin. *P < 0.05, **P < 0.01. (**C**) Western blot analysis of downstream factors on QGY7703 and PLC8024 vector and AKR7A3 transfected cells. EMT markers include fibronectin and Snail. Signaling pathways include c-Jun, NF-κB and ERK pathways, as demonstrated by down-regulated p-c-Jun, p-NF-κB and p-ERK in AKR7A3 overexpressed cells. β-actin was used as loading control.

To investigate the effects of AKR7A3 on chemoresistance in HCC, cisplatin—a chemotherapeutic drug—was used to treat 8024-AKR7A3 and H2M-sh3 cells together with their respective control cells. Cell viability assay (XTT) was performed on cells treated with gradient concentrations of cisplatin and vehicle control. Results showed that AKR7A3 overexpressed cells are more prone to apoptosis upon cisplatin treatment (*P < 0.05; **P < 0.01; ***P < 0.001) (Figure 5A), whereas AKR7A3 knockdown cells are more chemoresistant (*P < 0.05; **P < 0.01) (Figure 5B).

### AKR7A3 exerts tumor suppressive effects through attenuating ERK, c-Jun and NF-κB signaling pathways

In light of the confirmation of the tumor suppressive abilities of AKR7A3, the downstream signaling pathways were studied. Cell lysates of 8024-AKR7A3 and 7703-AKR7A3 along with their empty vector controls were harvested and subjected to western blot analysis. In both cell lines, the expression of two of the epithelial–mesenchymal transition (EMT) markers, including fibronectin and Snail, were detected to be inhibited by AKR7A3 overexpression (Figure 5C), which could partly explain the effects of AKR7A3 overexpression on migration and invasion of HCC cells. Overexpression of AKR7A3 was further shown to attenuate three important signaling pathways controlling liver cancer development, including ERK, c-Jun and NF-κB signaling pathways, as demonstrated by the significant lower levels of p-ERK, p-c-Jun and p-NF-κB levels in AKR7A3 overexpressed cells than that in the control cells (Figure 5C).

## DISCUSSION

Hepatocellular carcinoma has long been considered one of the most lethal cancers worldwide with poor prognosis [1]. The elucidation of the genetic causes of HCC onset and progression has always been extensively pursued but is still far from complete. AKR7A3 is one of the key enzymes in the reduction of aflatoxin B1, a strong hepatocarcinogen [9]. There are mounting evidence supporting the critical function of this gene in normal liver processes.

Here, we reported that AKR7A3 acts as a potent tumor suppressor in hepatocellular carcinoma. AKR7A3 was not only found to be frequently down-regulated in HCC (Figure 1A), but also demonstrated strong clinical significance. The association between AKR7A3 expression and overall survival rate of HCC patients indicated the importance of AKR7A3 in preventing HCC onset and progression. Our data also showed the lower AKR7A3 expression being correlated with elevated serum AFP level, assuring the potential of AKR7A3 as a marker for HCC prediction and diagnosis.

Bisulfite genomic sequencing and methylation-specific PCR revealed promotor hypermethylation of AKR7A3, which partly explains the AKR7A3 down-regulation in HCC. Our data showed that the promoter region of AKR7A3 low expression cell lines (QGY7703 and PLC8024) were much more severely methylated than those of AKR7A3 high expression cell lines (97L and H2M) (Figure 2B and 2C). Chromosome 1p has been found to frequently harbor deletions of genetic materials in HCC [5]. AKR7A3 being located in this region hints deletion of this gene in HCC, which gives rise to its down-regulation. Loss of heterozygosity analysis on HCC patient DNA samples revealed the frequent deletion of AKR7A3, indicated by the high percentage of patients having allele loss in HCC tissue (43% of AKR7A3 down-regulated HCC samples showed LOH; Table 3).

The tumor suppressive functions of AKR7A3 were explored both *in vitro* and *in vivo*. Foci formation and soft agar assays determine the abilities of cells to grow into colonies under anchorage-dependent and anchorage-independent conditions, respectively. In both assays, AKR7A3 overexpression strongly inhibited colony growth, while AKR7A3 knockdown promoted colony formation. For migration and invasion abilities, AKR7A3 overexpressed cells migrated and invaded in much slower manners, while AKR7A3 knockdown cells migrated and invaded faster (Figure 4D and 4E). For *in vivo* tumor formation assay, PLC8024 cell line overexpressed with AKR7A3 could not form tumor subcutaneously, while control cells all formed tumors (Figure 3F). The effect of AKR7A3 on the chemoresistance of HCC was also tested. AKR7A3 overexpressed and control cells were treated with cisplatin and cell viability assay was performed. Cells overexpressed with AKR7A3 demonstrated much lower chemoresistence than control cells (Figure 5A). All of the above assays supported our assumption that AKR7A3 strongly suppresses the oncogenic abilities of cancer cells.

In order to understand the underlying mechanisms of AKR7A3 tumor suppressive effects, the activity of certain important signaling pathways in liver cancer development, including MAPK, c-Jun and NF-κB, were detected by western blot. ERK1 and ERK2 were found to be constitutively activated in various types of human tumor tissues [12]. They play pivotal roles in promoting cell proliferation and metastasis in human cancers [13]. Our data showed that AKR7A3 potently inactivates ERK phosphorylation, which could in part explain the strong tumor suppressive effects of AKR7A3. c-Jun is a proto-oncogene and a transcription factor. It has been reported that c-Jun protects hepatocytes from apoptosis through antagonizing p53 functions [14]. NF-κB is a protein complex that expresses extensively in animal cell types. In many types of cancers, NF-κB activates the transcription of anti-apoptotic proteins, leading to uncontrolled cell proliferation and tumor growth [15]. We found that AKR7A3 overexpressed cells have lower c-Jun and NF-κB activities, which could explain our data of AKR7A3 antagonizing cell growth and sensitizing HCC cells to chemotherapy drugs.

Collectively, our data demonstrated frequent down-regulation and clinical significance of AKR7A3 in HCC, as well as its strong tumor suppressive functions and inhibition of chemoresistance, which could be attributed to the attenuation of ERK, c-Jun and NF-κB signaling pathways. This report is the first to demonstrate the tumor suppressive functions of AKR7A3 in HCC, which gene was rarely studied in human cancers. The strong suppression of ERK, c-Jun and NF-κB signaling pathways by AKR7A3 reflects the multiple and complex molecular inhibition effects of this gene on the development and progression of HCC. This finding will provide new insight into the pathogenesis of HCC, potentially help develop new therapeutic strategies against this lethal disease.

## MATERIALS AND METHODS

### Clinical HCC tissue samples and cell lines

Clinical HCC tumor and adjacent non-tumor tissues were collected from Sun Yat-Sen University Cancer Center (Guangzhou, China). All HCC patients gave written informed consent on the use of clinical specimens for medical research. The collection of human tissues was approved by the Committees for Ethical Review of research involving human subjects of Sun Yat-Sen University (Guangzhou, China) and the Institutional Review Board of the University of Hong Kong.

Immortalized human liver cell line (LO2) and HCC cell lines (QSG7701, BEL7402, QGY7703, PLC8024, HepG2 and Huh7) were obtained from the Institute of Virology, Chinese Academy of Medical Sciences (Beijing, China). MHCC-97L was obtained from the Liver Cancer Institute of Fudan University (Shanghai, China). H2M was previously established in our laboratory. The 293FT cell line was purchased from Invitrogen. All cell lines used in this study were regularly authenticated by morphological observation.

### Bisulfite genomic sequencing (BGS) and methylation-specific PCR (MSP)

Genomic DNA was extracted and followed by bisulfite treatments using EpiTECT Bisulfite Kit (Qiagen). The CpG islands of AKR7A3 were predicted using software online (MethPrimer v1.1 beta, Li Lab, Department of Urology, USCF). Primers were designed for PCR amplification of chosen CpG islands. Primer specificity was examined by DNA gel electrophoresis. The PCR products were subsequently cloned into pMD18-T vector and transformed into DH5α competent cells. 5 to 10 colonies were randomly picked and sequenced. MSP was used to test the methylation status of CpG islands on a larger cohort of samples. Primers were designed based on the sequencing results above and validated by DNA gel electrophoresis. Sequences of primers were listed in Supplementary Table S1.

### Loss of heterozygosity analysis

Primers flanking two SNP positions (rs1738025 and rs2231198) were designed for amplifying DNA sequences of around 800 bp. PCR products were subjected to sequencing. The absence of dual peaks in tumor samples whose paired non-tumor sample harbors dual peak at SNP positions were considered LOH samples.

### AKR7A3 overexpression and knockdown

The coding region sequence of AKR7A3 was amplified by PCR and cloned into pLenti6/V5-TOPO vector (Invitrogen). Empty vector or AKR7A3 constructs were co-transfected with the ViraPower Lentiviral packaging plasmids (Invitrogen) into 293FT cells. Supernatant containing lentivirus was added to QGY7703 and PLC8024 cells. Blasticidin (Invitrogen) was added to the cells for screening for stably transduced cells after 24 hours.

For knockdown of AKR7A3, plasmids of two short hairpin RNAs targeting AKR7A3 and scrambled control were purchased from GeneCopoeia (Rockville). These plasmids were packed into virus and transduced into MHCC-97L and H2M cell lines. Puromycin was used to screen for stably transfected cells.

### Quantitative real-time PCR

Total RNA of cell lines and clinical HCC tissues were isolated using TRIzol reagent (Invitrogen). Complementary DNA was synthesized using PrimeScript RT Master Mix (Takara). The expression levels of genes were detected with SYBR Green PCR Kit (Roche) using ABI Prism 7900 System. Specificity of primers were validated by DNA gel electrophoresis. Primer efficiency and sensitivity were further tested and validated by standard curves and melt curves in qRT-PCR. Sequences of the respective primers were listed in Supplementary Table S1. Relative expression differences were calculated using the 2^−ΔΔCt^ method. 18s was used as endogenous control.

### *In vitro* and *in vivo* tumorigenicity functional assays

Foci formation assays were conducted by seeding 1000 cells/well in a 6-well plate in triplicates and colonies were counted after 2 to 3 weeks. In soft agar assay, 5000 cells/well were suspended in 0.35% agarose in DMEM/10% FBS in 6-well plates as the upper layer, with 0.5% agarose in DMEM/10% FBS being the bottom layer. Cells in agarose were allowed to grow for 2 to 3 weeks and the numbers of colonies formed in each well were counted. In migration and invasion assays, cells were seeded in cell culture inserts or invasion chambers in serum free DMEM medium, with the bottom of the chambers immersed in DMEM/10% FBS. After 48 to 72 hours, migrated or invaded cell were counted under microscope. For *in vivo* tumor formation assay, PLC8024-AKR7A3 and control cells were subcutaneously injected into the right and left dorsal flanks of 5 nude mice. Tumor sizes were monitored. All animal procedures were approved by Committee on the Use of Live Animals in Teaching and Research (CULATR), the University of Hong Kong.

### Cisplatin treatment and cell viability assay

For chemotherapy-induced cytotoxicity, 5000 cells/well were seeded in 96-well plates and treated with cisplatin for 48 hours. Cell viability was measured by XTT. The percentage of viable cells was determined using the following formula: cell viability (%) = A_492/630_ of treated cells / A_492/630_ of control cells.

### Western blot

Protein samples were denatured and separated in SDS-PAGE gel and transferred to PVDF membranes. The membranes were blocked with 5% non-fat milk and incubated with primary antibodies at 4^°^C overnight and HRP-conjugated secondary antibodies for 1 hour at room temperature. The chemiluminescence signals were visualized by exposing the ECL-incubated membranes to X-ray films.

### Statistical analysis

Statistical analysis was performed by using SPSS version 20.0 (SPSS, Inc.) and GraphPad Prism 5.0 (GraphPad Software, Inc.). Independent Student's *t*-test was applied to compare the mean values of two groups. Error bars represent SD values. The clinicopathological features in patients with and without AKR7A3 down-regulation were compared using Pearson's chi-square test for categorical variables. Kaplan-Meier plots and Log Rank tests were used for overall survival and disease-free survival analysis. Statistical significance was defined as *P* ≤ 0.05.

## SUPPLEMENTARY MATERIALS FIGURES AND TABLE


