# Supplementary material for: AKR7A3 suppresses tumorigenicity and chemoresistance in hepatocellular carcinoma through attenuation of ERK, c-Jun and NF-κB signaling pathways
Source: Oncotarget. 2016 Oct 18;8(48):83469–79. doi: 10.18632/oncotarget.12726 (PMC5663529; doi:10.18632/oncotarget.12726)
Supplement: Supplementary file 1 [file oncotarget-08-83469-s001.pdf]

AKR7A3 suppresses tumorigenicity and chemoresistance in hepatocellular carcinoma through attenuation of ERK, c-Jun and NF-κB signaling pathways

Supplementary Materials

Supplementary Table S1: Sequences of primers used in this study

Primers		Sequence (5′-3′)	Product Size (bp)	T_m (°C)
**Primers for real-time quantitative PCR (qPCR)**				
qRT-AKR7A3	Forward	GCCCACTGTGTACCAGGGCATGTA	171	60
	Reverse	CCCACGGGCTGTTTCCCATTCT		
qRT-18S	Forward	CTCTTAGCTGAGTGTCCCGC		
	Reverse	CTGATCGTCTTCGAACCTCC		
**Primers for gene cloning**				
CLO-AKR7A3	Forward	CACCTGGTGCTTCCGACCG	1052	60
	Reverse	GCGGAAGTAGTTGGGACATT		
**Primers for loss of heterozygosity (LOH) analysis**				
rs1738025	Forward	GTGAGTAACAAAAGATGTTACAGAAGAGCC	326	60
	Reverse	ATATGGGATACAAGAGATCCCAAATCTCAG		
rs2231198	Forward	TTGCTCTTGCAGAGGGTACAGAT	355	63
	Reverse	GAGTGGACCTCTTCTACCTGCATA		
**Primers for bisulfite genomic sequencing (BGS) and methylation-specific PCR (MSP) analysis**				
BGS-AKR7A3	Forward	TTATTTTTGGATTTTTTTATTTTTGG	536	53
	Reverse	AAAAACCACTATTACCTCTACAATC		
M-AKR7A3	Forward	CGTATGGACGCGTTTATTAGC	165	56
	Reverse	AAAACCACTATTACCTCTACAATCG		
U-AKR7A3	Forward	GTTGTATGGATGTGTTTATTAGTGT	165	56
	Reverse	AACCACTATTACCTCTACAATCACT		

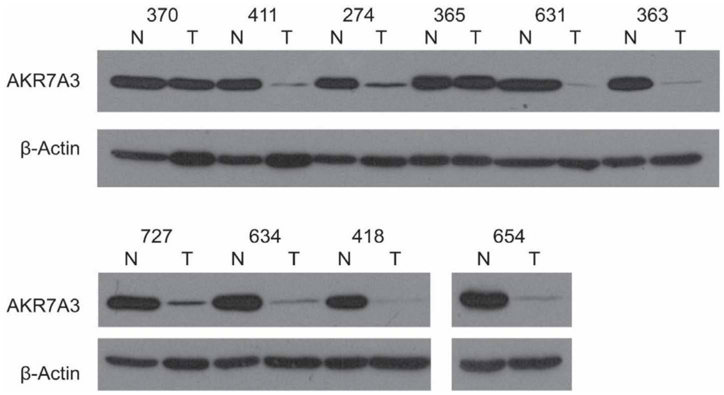

Supplementary Figure S1: Western blot analysis of AKR7A3 on 10 pairs of HCC patient samples. AKR7A3 protein expression dramatically decreased in HCC tissues as compared with adjacent non-tumor tissues.

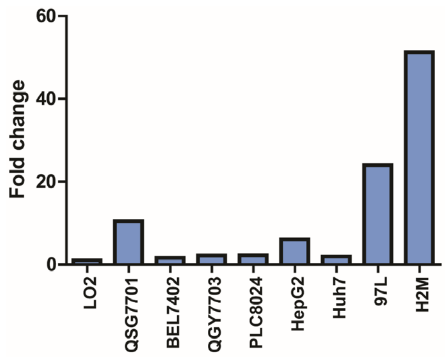

Supplementary Figure S2: Endogenous AKR7A3 expression levels in immortalized liver cell lines and HCC cell lines as detected by qRT-PCR.
